# Mitigating Blue-Light Risk in Display-Based Digital Therapeutics: A Practical Framework to Support Clinical Efficacy

**DOI:** 10.3390/jcm15041371

**Published:** 2026-02-09

**Authors:** Wonki Hong

**Affiliations:** Division of Digital Healthcare, Yonsei University Mirae Campus, Wonju 26493, Republic of Korea; wkhong@yonsei.ac.kr

**Keywords:** digital therapeutics, blue light exposure, display optics, circadian rhythm, melatonin suppression

## Abstract

Display-driven optical stimuli underpin a major class of clinically validated digital therapeutics (DTx) now expanding from neuropsychiatric disorders to chronic diseases. The display’s optical characteristics—spectral power distribution, luminance, contrast, and temporal modulation—therefore define the delivered dose of these software-based interventions. In this context, blue-rich emission in the 450–480 nm band, particularly with evening exposure, can suppress melatonin via melanopsin-mediated intrinsically photo-sensitive retinal ganglion cell (ipRGC) pathways and perturb circadian timing, potentially attenuating therapeutic efficacy. This review summarizes clinical evidence for display-enabled DTx across major indications and synthesizes mechanistic and experimental data linking blue light to sleep and circadian disruption, with downstream mood, cognitive, cardiovascular, and metabolic effects, as well as increased risk of cancer and skin damage. This review distinguishes wavelength-dependent hazards by separating retinal photochemical risk in the roughly 415–450 nm range from circadian-disruptive melanopic effects in the 450–480 nm range, informing spectrum optimization for therapeutic use. It then synthesizes mitigation strategies spanning display emitter spectrum engineering, optical filtering or conversion films, and software controls such as color temperature tuning, high-frequency dimming, metameric spectrum design, and personalized circadian lighting. The review concludes with design, prescription, and standards considerations to align display output with therapeutic intent.

## 1. Introduction

Software-based digital therapeutics (DTx), developed based on medical evidence, are increasingly used to deliver personalized interventions via digital platforms and applications [1,2,3]. These solutions are widely applied to mental health programs, including smartphone-based psychological interventions and internet-delivered cognitive behavioral therapy for anxiety and depression [4,5]. Digital therapeutics are software-based medical interventions that aim to improve health outcomes by inducing cognitive and behavioral changes rather than relying primarily on pharmacological mechanisms [6,7]. In practice, they are delivered through digital modalities such as AI-enabled conversational agents and app-based therapeutic programs, and they may incorporate engagement strategies including gamification [8,9]. This approach is widely utilized for mental health conditions, including insomnia [10], attention deficit hyperactivity disorder (ADHD) [11], and anxiety/depression [12,13]. It has also been explored in cardiovascular care as digital therapeutics continue to expand in cardiology [14]. DTx enables personalized treatment by providing individualized protocols based on artificial intelligence and big data analysis. This approach supports self-management without time and location constraints, reduces unnecessary hospitalizations and medication use, and contributes to reducing medication side effects and healthcare costs [15,16,17]. However, DTx raises concerns regarding a diminished sense of reality due to excessive dependence, the risk of developing new forms of addiction, and potential issues such as privacy breaches and adverse effects stemming from their software-based nature [18,19].

**Figure 1:**
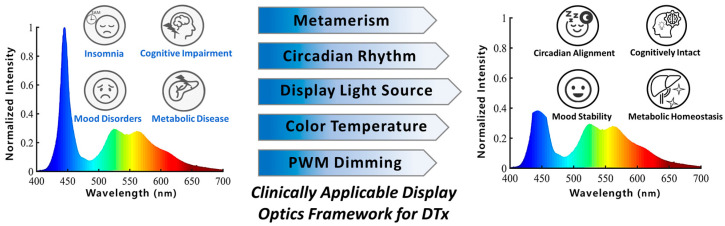
Schematic illustration of the potentially detrimental effects of blue light emitted from digital displays on the therapeutic efficacy of DTx (**left**) and its enhancement through advanced technological strategies to mitigate blue light exposure and optimize wavelength parameters for clinical applications (**right**). The blue-to-red color gradient indicates wavelength across the visible spectrum (≈400–700 nm), and the plotted curves represent normalized spectral intensity.

In particular, the severity of the display blue light issue needs to be reassessed. Since digital therapeutics (DTx) are designed around digital platforms such as smartphones, virtual reality (VR), augmented reality (AR), smartwatches, and tablets, displays are an indispensable component. Because displays are a major and controllable source of short-wavelength light at night, evidence from nighttime device use and broader light-at-night exposure is directly relevant to display-enabled DTx. Paradoxically, light exposure patterns related to nighttime device use and light at night are associated with poorer sleep quality and adverse mood and cognitive outcomes [20]. Experimental and review evidence further links short-wavelength or nighttime light exposure to metabolic dysregulation and increased cardiometabolic risk, including obesity, impaired glucose homeostasis, and atherosclerosis [21,22]. In other words, exposure to blue light from a display may contradict the therapeutic purpose of digital therapeutics, potentially undermining their effectiveness. This review examines the adverse impact of blue-rich display emission on display-enabled digital therapeutics and summarizes hardware- and software-level mitigation strategies that may help align display output with therapeutic intent, as illustrated in Figure 1. Figure 1 schematically contrasts a blue-rich display spectrum on the left, associated with sleep and circadian disruption and downstream risks, with a mitigated spectral profile on the right designed to support circadian alignment and physiological stability. The center of Figure 1 summarizes the main controllable levers discussed in this review, including metameric spectrum design, circadian-informed output control, display light source engineering, color-temperature tuning, and pulse-width modulation (PWM)-based dimming. Together, the figure provides a unified framework connecting wavelength-dependent hazards to practical mitigation strategies for display-enabled DTx. This review explores wavelength-dependent issues of display-emitted blue light that are directly relevant to display-enabled digital therapeutics, in which circadian disruption may undermine therapeutic intent. By distinguishing the circadian-relevant band associated with melatonin suppression (450–480 nm) from shorter-wavelength regions implicated in retinal photochemical hazard, it outlines spectrum-optimization directions for therapeutic use and summarizes practical mitigation strategies. The review is structured to first summarize recent clinical progress of DTx across major indications (Section 2), then synthesize evidence linking blue-light exposure to circadian disruption and downstream risks (Section 3), and finally synthesize hardware- and software-level approaches and design/standards considerations to align display output with therapeutic intent (Section 4, Section 5 and Section 6). Relevant literature was identified through targeted searches of major bibliographic databases using keywords related to display spectra, melanopic metrics, circadian disruption, and digital therapeutics and prioritized based on relevance to display-enabled DTx.

## 2. Recent Progress in Digital Therapeutics for Various Disorders

Digital therapeutic software solutions are utilized to treat various sleep disorders, demonstrating particularly notable efficacy in managing insomnia. Digital cognitive behavioral therapy for insomnia (dCBT-I) has demonstrated measurable improvement in the Insomnia Severity Index, sleep efficiency, sleep onset latency, and nocturnal awakenings through its core components, including sleep education, sleep diaries, and relaxation techniques [23,24]. The methodology of dCBT-I offers advantages in terms of accessibility and cost-effectiveness, yielding substantial benefits not only in enhancing sleep quality but also in fostering lifestyle modifications, including greater awareness of sleep hygiene principles. Even unguided dCBT-I interventions can achieve significant clinical efficacy while addressing the shortage of trained therapists, indicating strong potential for practical implementation [25]. When applied to patients with sleep apnea, dCBT-I can strengthen adherence to continuous positive airway pressure (CPAP) therapy and contribute to better sleep outcomes in individuals experiencing nightmares [26,27]. Additionally, digital therapeutics have demonstrated clinically meaningful benefits and effectiveness in patients exhibiting symptoms of depression and anxiety. AI-driven conversational apps and other smartphone-based digital mental health interventions have played a role in expanding accessibility to depression management among populations such as perinatal women and adolescents, even during crises like COVID-19, by leveraging a self-guided CBT model [28]. Digital cognitive behavioral therapy (dCBT) has demonstrated long-term advantages in patient groups with high levels of engagement, while guided and unguided approaches have exhibited comparable effectiveness in individuals with mild depression [29,30]. Moreover, digital interventions, including app-based and internet-delivered CBT (iCBT) for Generalized Anxiety Disorder (GAD), have demonstrated significant efficacy in alleviating anxiety not only in controlled trials but also in real-world clinical settings [31,32]. Multifaceted studies have shown that internet-based Cognitive Behavioral Therapy (iCBT) yields symptom relief comparable to face-to-face therapy for social anxiety disorder, consistently demonstrating substantial and stable therapeutic effects [33,34]. Virtual reality (VR)-based online exposure therapy effectively reduces fear and avoidance behaviors in social interactions by minimizing spatial and temporal constraints while alleviating the burden associated with face-to-face therapy compared to traditional treatments.

Digital therapeutics (DTx) utilizing personalized interventions promote neuroplasticity in neurological disorders, exerting positive effects on neurodegenerative processes and strengthening cognitive function and memory. The implementation of immersive cognitive training programs using VR/AR for patients with mild cognitive impairment (MCI) has resulted in advancements in neuropsychological indicators such as attention, spatial perception, and memory while also demonstrating advantages in reinforcing motivation and social engagement. For instance, video-based exergames have been shown to boost attention and processing speed (SDMT, PASAT) in patients with multiple sclerosis, while multimedia-assisted digital reminiscence therapy (RT) has been found to support memory, attention, behavioral and psychological symptoms, depression, and overall well-being in patients with Alzheimer’s disease and dementia [35,36,37]. Additionally, digital cognitive training administered to patients with neurological disorders such as stroke and traumatic brain injury (TBI) has yielded measurable gains in attention, executive function, and language abilities compared to the control group [38,39]. Finally, clinical trials of digital cognitive training and the therapeutic software MindPro1 (V1.1) for children with attention-deficit/hyperactivity disorder (ADHD) have demonstrated significant refinements in core cognitive domains, including working memory, attention, and response inhibition, with additional synergistic effects observed when combined with pharmacotherapy [40,41].

Digital therapeutic interventions have the potential to transform the prevention and management of cardiovascular diseases, including hypertension and stroke, by fostering non-pharmacological lifestyle modifications and medication adherence. In a randomized controlled trial (RCT), hypertensive patients receiving digital therapeutics exhibited reductions in systolic blood pressure. Notably, when combined with AI-driven coaching, digital therapeutics supported non-pharmacological lifestyle modifications, including reduced salt intake, increased physical activity, and weight loss, reinforcing self-efficacy in hypertension self-management [42,43,44]. A home-based cardiac rehabilitation program incorporating home training, teleconsultation, and gamification significantly enhanced exercise adherence and overall well-being, demonstrating progress comparable to or superior to that of hospital-based rehabilitation in terms of cardiorespiratory function [45,46,47]. A text message-based program facilitated lifestyle adjustments, including increased vegetable and fruit consumption, in patients with acute coronary syndrome, demonstrating benefits in lowering low-density lipoprotein (LDL) cholesterol and blood pressure [48]. Diabetes, a leading metabolic disease, also presents substantial potential for digital therapeutics through patient-centered personalized management strategies. Numerous studies have demonstrated that mobile app-based self-management programs for patients with diabetes contribute to substantial reductions in glycemic markers, including glycated hemoglobin (HbA1c), fasting blood glucose, and insulin resistance [49,50]. Digital therapeutics (DTx), which integrate behavioral medicine and advanced digital technology, have been shown to facilitate weight loss, BMI reduction, and body fat decrease across various age groups, including children and adolescents, yielding superior outcomes compared to control groups [51,52]. Additionally, recent applications of digital therapeutics (DTx) in patients with metabolic dysfunction-associated steatotic liver disease (MASLD) and non-alcoholic fatty liver disease (NAFLD) have demonstrated clinical efficacy by substantially reducing liver enzyme levels (ALT, AST) [53,54].

DTx applications have demonstrated significant clinical outcomes across various stages of cancer, from prevention and treatment to supportive care. DTx programs designed for cancer prevention effectively facilitate health behavior changes by reducing smoking and alcohol consumption, promoting physical activity, and increasing cancer-related knowledge [55]. A randomized controlled trial (RCT) involving patients with advanced cancer demonstrated that digital therapeutic interventions substantially enhanced pain management and health-related quality of life (HRQOL), reduced emergency room and outpatient visits, and achieved statistically significant benefits in survival metrics compared to conventional treatment groups [56]. DTx helps mitigate postoperative and chemotherapy-related complications through multidimensional symptom management while optimizing treatment adherence and mitigating adverse effects in supportive cancer care, ultimately leading to lower readmission rates and optimized quality of life [57,58]. For example, integrating digital therapeutics (DTx) with clinical decision support systems (CDSS) has been reported to positively impact self-management capabilities, psychological support, and lifestyle modifications in patients with breast cancer [59].

Multiple digital intervention studies have demonstrated advancements in the treatment of chronic skin conditions, such as atopic dermatitis and psoriasis. Internet-based cognitive behavioral therapy (iCBT) has been shown to alleviate pruritus and lower EASI scores in patients with atopic dermatitis while facilitating real-time tracking and management, ultimately optimizing clinical outcomes and quality of life [60,61,62]. Digital therapeutics can serve as a complementary treatment for psoriasis by mitigating psychosocial distress, reducing depression and anxiety, and enhancing overall well-being and self-efficacy [63]. Additionally, prescription digital therapeutics (PDTx) support treatment adherence and quality of life by enabling real-time tracking and management of complications associated with chronic dermatological conditions, including skin cancer, acne, rashes, pain, and psychological stress [64].

## 3. The Challenge of DTx: Detrimental Health Effects of Display Blue Light

Blue-light exposure in the 450–480 nm range activates melanopsin-expressing intrinsically photosensitive retinal ganglion cells and can disrupt circadian timing; such circadian disruption has been associated with adverse outcomes across sleep, mood, cognitive, and cardiometabolic domains and has been discussed in relation to broader long-term health risks.

**Figure 2:**
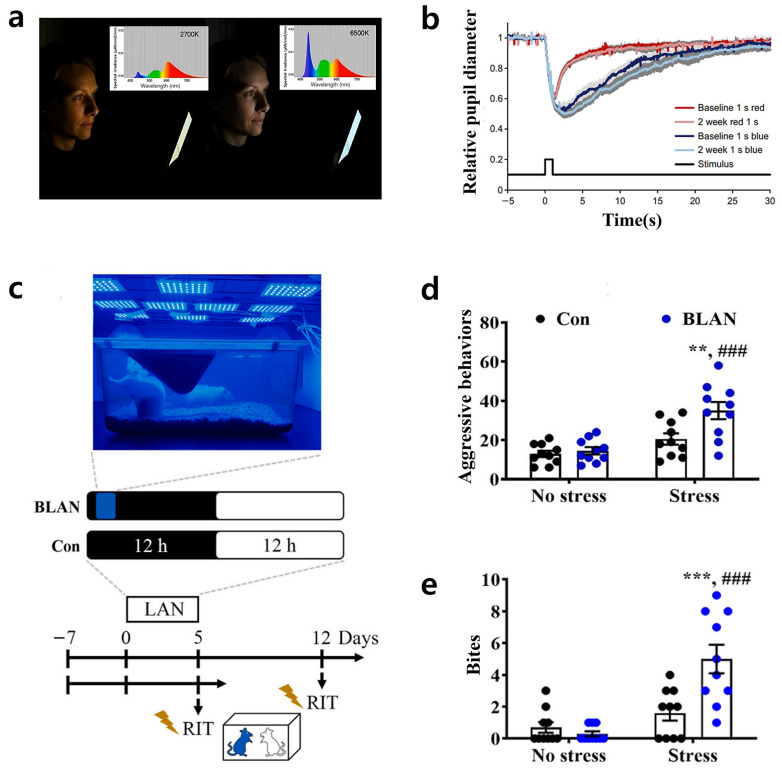
Increased risk of insomnia and exacerbation of stress-reactive aggressive behavior associated with blue light exposure. (**a**) Standard smartphone display with no filter (right panel): Prominent short-wavelength blue peak and a key contributor to potential blue light hazards. In the spectra, the blue-to-red color gradient denotes wavelength across the visible range. Reprinted from Ref. [66] with permission from W.B. Saunders Co., Ltd. (**b**) Blue light exposure: Leads to marked pupil constriction, indicative of strong physiological impact. The light-grey shading indicates the 95% confidence interval around the baseline mean. Reprinted from Ref. [68] with permission from Wiley. (**c**) A rat model exposed to blue light at night. Reprinted from Ref. [72] with permission from Elsevier. (**d**) Exacerbation of aggressive behaviors under stress following BLAN (Blue Light at Night) exposure. ** *p* < 0.01 vs. Con + Stress; ### *p* < 0.001 vs. BLAN + No stress. Reprinted from Ref. [72] with permission from Elsevier. (**e**) BLAN exposure under stress: Significant rise in anxiety-like behavior. *** *p* < 0.001 vs. Con + Stress; ### *p* < 0.001 vs. BLAN + No stress. Reprinted from Ref. [72] with permission from Elsevier.

Exposure to blue light from displays, as shown in Figure 2a, significantly impairs sleep quality, reduces overall sleep duration, and is associated with lower sleep efficiency [65,66,67]. This light activates melanopsin-containing photoreceptors in the retina, specifically intrinsically photosensitive retinal ganglion cells (ipRGCs), leading to melatonin suppression and subsequent autonomic nervous system stimulation, which may induce insomnia. The circadian rhythm governs the sleep–wake cycle by modulating melatonin secretion, suppressing it during the day and promoting it at night. However, continuous exposure to blue light physiologically constricts the pupil, as shown in Figure 2b, suppresses melatonin secretion, prolongs sleep latency, and can induce Delayed Sleep–Wake Phase Disorder (DSWPD), leading to impaired daytime attention performance [68,69,70,71]. Additionally, blue light is implicated in the development of mental health disorders, including stress, depression, and bipolar disorder. Nocturnal exposure can increase Brain-Derived Neurotrophic Factor (BDNF) expression in the basolateral amygdala (BLA), potentially amplifying aggressive behavior in response to stress, as illustrated in Figure 2c–e [72]. This BLA-BDNF activation disrupts the amygdala’s regulation of fear and threat responses, leading to excessive stress reactions and potentially inducing behavioral and neurobiological changes. Furthermore, it suppresses melatonin secretion from the pineal gland, disturbs the sleep–wake cycle, and desynchronizes circadian gene expression, which may contribute to depression and mood disorders [73,74]. Studies have shown that blue light-blocking therapy can improve sleep quality and circadian rhythm regulation in patients with bipolar disorder [75,76]. These findings suggest that short-wavelength light-blocking strategies may serve as an effective adjunctive treatment for mood disorders by alleviating melatonin suppression and mood disturbances caused by nocturnal blue wavelengths.

**Figure 3:**
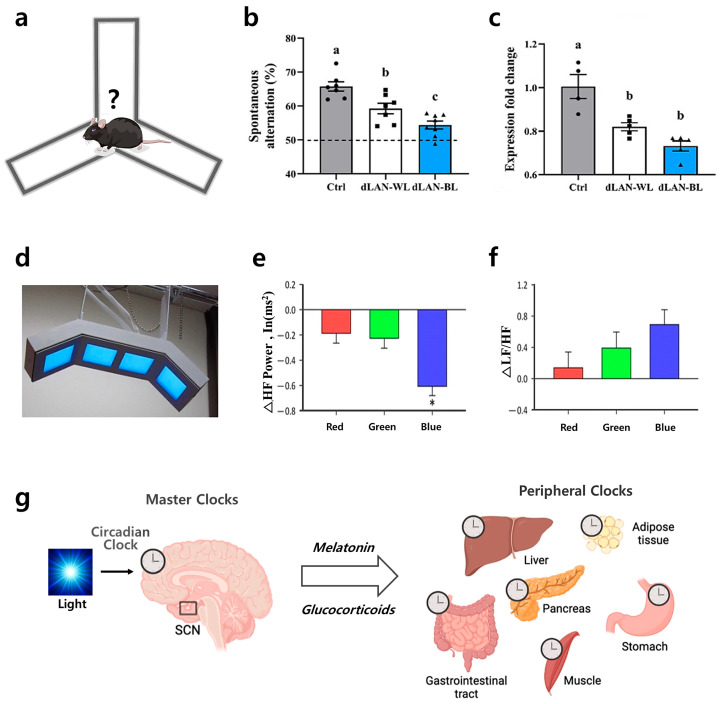
Cognitive impairment, cardiovascular disease, and metabolic disease associated with blue light. (**a**) Diagram illustrating the Y-maze layout. Reprinted from Ref. [77] with permission from MDPI. (**b**) dLAN exposure (WL and BL): Significant reduction in spontaneous alternation performance (Control > dLAN-WL > dLAN-BL). Reprinted from Ref. [77] with permission from MDPI. Values not sharing a common superscript letter (a,b,c) differ significantly at *p* < 0.05; those with the same letter (a,b,c) do not differ significantly (*p* ≥ 0.05). (**c**) dLAN exposure: Significant decrease in neurotrophic factor BDNF expression, most pronounced with blue light. Marker shapes denote individual data points (one animal per marker) and are used only for visual distinction. Reprinted from Ref. [77] with permission from MDPI. (**d**) OLED lighting apparatus used to provide the controlled blue light exposure for the experiments shown in (**e**,**f**). Reprinted from Ref. [86] with permission from BMC. (**e**) Harmful effects of blue light: Pronounced reduction in ΔHF power under blue light compared to red and green conditions. Reprinted from Ref. [86] with permission from BMC. * Significantly different from values for red and green with multiple comparisons (*p* < 0.05). (**f**) Blue light hazard: Statistically significant elevation of ΔLF/HF ratio under blue light relative to red and green exposure. Reprinted from Ref. [86] with permission from BMC. (**g**) Hierarchical circadian network: The light-entrained suprachiasmatic nucleus (SCN) master clock coordinates peripheral clocks influenced by feeding/exercise cues and hormonal feedback. Reprinted from Ref. [89] with permission from Elsevier.

Blue light spectrum also affects cognitive function and learning. Figure 3b,c illustrate that mice subjected to continuous dim blue light at night (dLAN-BL) exhibit increased plasma corticosterone levels, neuronal loss, and accelerated impairments in spatial learning when assessed using the maze experiment depicted in Figure 3a [77]. Conversely, studies on patients with insomnia show that blocking blue light at night enhances processing speed and working memory. Additionally, the use of electronic devices just before sleep disrupts mood regulation and negatively impacts sleep quality, memory, and concentration, ultimately impairing overall cognitive function [78,79]. Furthermore, light at night interferes with circadian gene expression and induces neurophysiological changes, potentially contributing to neurodegenerative diseases such as Alzheimer’s disease [80]. Moreover, experiments in adult fruit flies show that prolonged exposure to blue wavelengths accelerates aging and induces retinal and neuronal degeneration, shortening lifespan [81]. Chronic blue light exposure disrupts the normal circadian rhythm of the cardiovascular system, leading to rigid regulation of blood pressure and heart rate and impairing the body’s ability to maintain homeostasis [82]. It reduces sleep duration and disturbs blood pressure variability, exacerbating cardiovascular risks associated with sleep deprivation [83]. For example, sustained exposure to this light may increase blood pressure, elevate heart rate, worsen insulin resistance, and impair vascular function, thereby raising the risk of cardiovascular diseases [84,85]. Furthermore, blue light emitted by organic light-emitting diode (OLED) lighting devices, as illustrated in Figure 3d, affects the autonomic nervous system, altering heart rate variability (HRV) through vagal nerve inhibition, as demonstrated in Figure 3e,f [86,87].

Blue light impacts various metabolic processes in the body, particularly causing imbalances in the gut microbiome, insulin secretion, fatty liver, and obesity. Figure 3g depicts the hierarchical circadian network where the light-synchronized SCN master clock regulates peripheral clocks in various organs under the influence of hormones and other cues. Exposure to this light at inappropriate times, such as during the evening or night, disturbs the circadian cycle, reduces gut microbiome diversity, and leads to an imbalance between harmful and beneficial bacteria, which can increase the risk of metabolic disorders [88,89]. Blue light exposure at inappropriate times induces fluctuations in blood glucose and insulin, disrupting metabolism, altering the circadian clock, raising plasma corticosterone levels, exacerbating hepatic oxidative stress, and potentially contributing to fatty liver development [90,91]. Moreover, blue light disrupts glucose homeostasis, contributing to the risk of obesity and other metabolic conditions [92,93,94].

**Figure 4:**
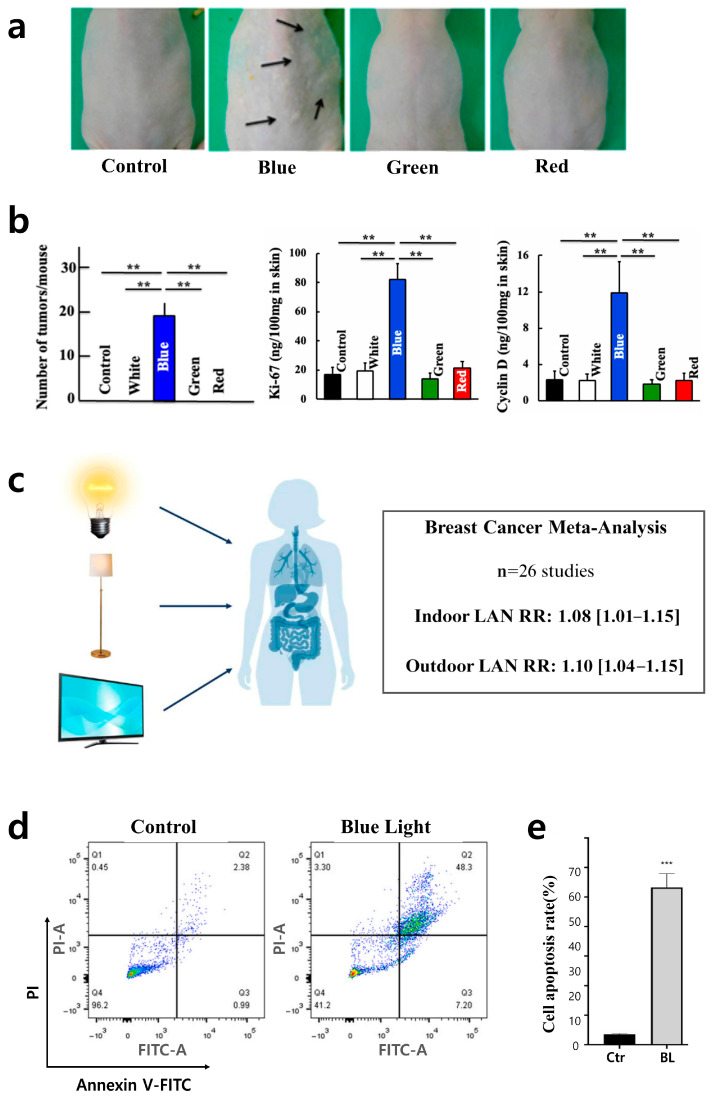
Potential cancer risk and skin diseases linked to blue light. (**a**) Notable pathological skin changes in the blue light exposure group relative to control and other tested light spectra. Arrows in the mouse exposed to blue light indicate representative tumor lesions. Reprinted from Ref. [97] with permission from MDPI. (**b**) Increased tumor multiplicity (left), elevated Ki-67 levels (middle), and higher Cyclin D levels (right) vs. control/other colors. Asterisks indicate statistical significance (** *p* < 0.01). Reprinted from Ref. [97] with permission from MDPI. (**c**) Light at Night (LAN) and breast cancer: Significant positive association for indoor LAN. Reprinted from Ref. [101] with permission from MDPI. (**d**) Flow cytometry analysis of apoptosis (Annexin V/PI) comparing control and blue light-exposed cells. Reprinted from Ref. [106] with permission from Elsevier. (**e**) Significant increase in the percentage of apoptotic cells from experiments shown in (**d**). (*** *p* < 0.001). Reprinted from Ref. [106] with permission from Elsevier.

Overall, blue light alters the gut environment and impairs energy metabolism, increasing the risk of obesity and potentially raising the risk of functional disorders. Artificial light, especially rich in the blue light spectrum, disturbs the biological clock, potentially leading to cancer development. Excessive exposure during nighttime suppresses melatonin secretion, disrupts the sleep–wake cycle, and interferes with hormonal balance and immune regulation, increasing the risk of cancer. Excessive blue light may contribute to liver and thyroid cancer pathogenesis through multiple mechanisms, including weakened immune surveillance, reduced antioxidant defense in tumors, increased cellular damage and DNA mutations, and disruption of circadian rhythm and metabolism [95,96,97]. For example, continuous exposure to one type of light uniquely caused visible skin damage and prominent formation of numerous growths linked to increased cell division, unlike other light conditions, as shown in Figure 4a,b. In addition, Figure 4c shows a significant correlation between nocturnal blue light exposure and prostate cancer, with direct associations with breast cancer extensively reported in both epidemiological and experimental research [98,99,100,101,102].

Additionally, it disrupts the function of clock genes in skin cells, which regulate cell regeneration, antioxidant activity, and collagen synthesis, thereby accelerating photoaging [103]. Blue light from digital devices penetrates deeply into the skin, promoting oxidative stress, inflammatory responses, and melanin synthesis, which weakens the skin’s defense mechanisms [104,105]. Figure 4d,e illustrate that blue light induces endoplasmic reticulum stress, leading to excessive autophagy, which promotes cell death [106]. Furthermore, it causes DNA damage in melanoma cells (B16F1), activating apoptosis-related pathways and worsening tissue degeneration [107]. The detrimental effects arising from display-based blue light exposure signify potential downsides of DTx; these effects, considered alongside the aforementioned benefits, are categorized into various disorder categories and summarized in Table 1.

## 4. Blue Light Mitigation Technologies for DTx Displays

The methods for reducing harmful blue light from displays can be approached from both hardware and software perspectives.

**Figure 5:**
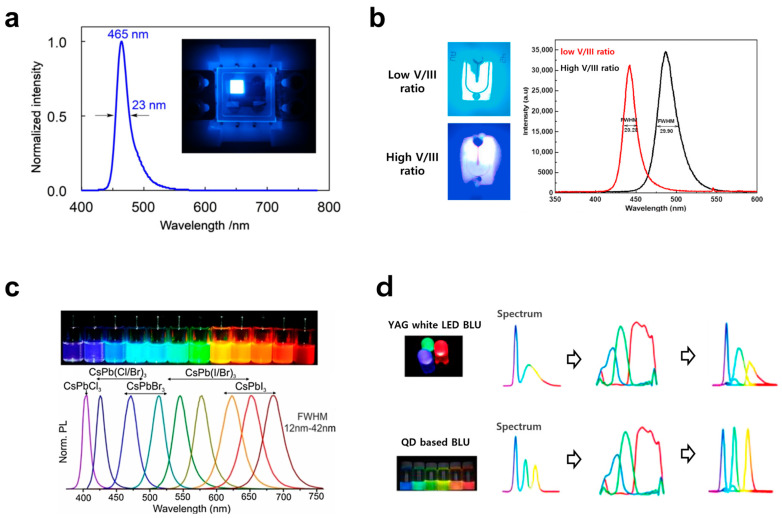
Blue light mitigation through display light source design. (**a**) Blue emission at a wavelength of 465 nm with an FWHM of 23 nm achieved through (N→O) atomic substitution in the high-performance blue emitter v-DABNA. Reprinted from Ref. [112] with permission from Nature Portfolio. (**b**) Control of emission wavelength, spectral bandwidth, and intensity according to the ratio of Group V and Group III elements in InGaN/GaN Multi-Quantum Wells. Reprinted from Ref. [115] with permission from Elsevier. (**c**) Wavelength shift due to the quantum confinement effect by controlling InP QD crystal size. Reprinted from Ref. [119] with permission from De Gruyter. (**d**) Mitigation of blue light hazards in QD-based BLUs through shifting the desired wavelength spectrum. In the spectra, colored curves denote different wavelength components. Reprinted from Ref. [119] with permission from De Gruyter.

The methods for reducing harmful wavelength ranges from a hardware perspective can be categorized into display light source design and optical filtering techniques. LCDs (Liquid Crystal Displays) that use backlighting have a technical characteristic in that they must select specific light spectra to optimize phosphor conversion efficiency. For this reason, it is difficult to avoid the harmful blue light peak in the 415–450 nm range. LCDs combine blue LEDs with red and green fluorescent materials to produce white LED backlighting. The phosphors typically used have the highest light efficiency around the 450 nm light range, making it easier to reduce power consumption and achieve stable light emission characteristics. As a result, the blue light within this range is inevitably emitted in high intensity. In contrast, OLED (Organic Light-Emitting Diode) displays, which use self-emissive technology where each subpixel emits light individually, can more easily avoid the 415–450 nm range [108,109,110,111,112,113]. For example, a specific blue emission wavelength is primarily achieved through elemental substitution within the organic light-emitting material itself, as shown in Figure 5a. Optimizing the performance and color purity for the blue subpixel also involves careful consideration of the doping concentration, synthesis method, layer structure, and arrangement. Due to the flexibility in designing the emission spectrum, OLED displays can reduce harmful blue light while also improving color reproduction.

Furthermore, micro-LED displays can effectively avoid the harmful short-wavelength light range by optimizing the design structure and adjusting the bandgap energy [114,115,116,117]. The blue chips for MicroLED are primarily based on InGaN epitaxial processes, and the peak emission wavelength can be shifted to longer wavelengths by adjusting the Indium concentration. This can be achieved through methods such as controlling the V/III ratio, as shown in Figure 5b. Alternately stacking the quantum well layers (InGaN) and barrier layers (GaN) in the epitaxial process optimizes the thickness of the quantum well, the barrier components, and the distribution of indium, allowing for modification of the emission bandwidth and range. Therefore, by reducing the intensity of blue light in the 415–450 nm range and increasing the intensity of longer wavelengths above 460 nm, InGaN/GaN-based blue LED devices can be designed to minimize harmful blue light emissions through precise spectrum control and quantum well structure optimization. The flexibility in light spectrum control and device structure design allows micro-LED displays to find a more favorable balance between human safety and light emission characteristics. Similar to OLEDs, QLED (Quantum Dot Electroluminescence) displays also employ a self-emissive technology in which each subpixel directly emits light, making them advantageous for shifting the blue emission wavelength [118,119,120,121]. By optimizing the particle size, material composition, and arrangement structure of the blue quantum dot pixels based on the quantum confinement effect, short-wavelength blue light in the 415–450 nm range can be effectively suppressed, and the emission spectrum can be theoretically shifted to the longer-wavelength region, as shown in Figure 5c. Fundamentally, controlling the emission spectrum of the device itself offers a significant advantage, as it allows for the reduction in harmful blue light while still achieving the desired color reproduction and efficiency, as shown in Figure 5d. However, conventional QLED TVs that use blue LEDs for backlighting are excluded from this category.

**Figure 6:**
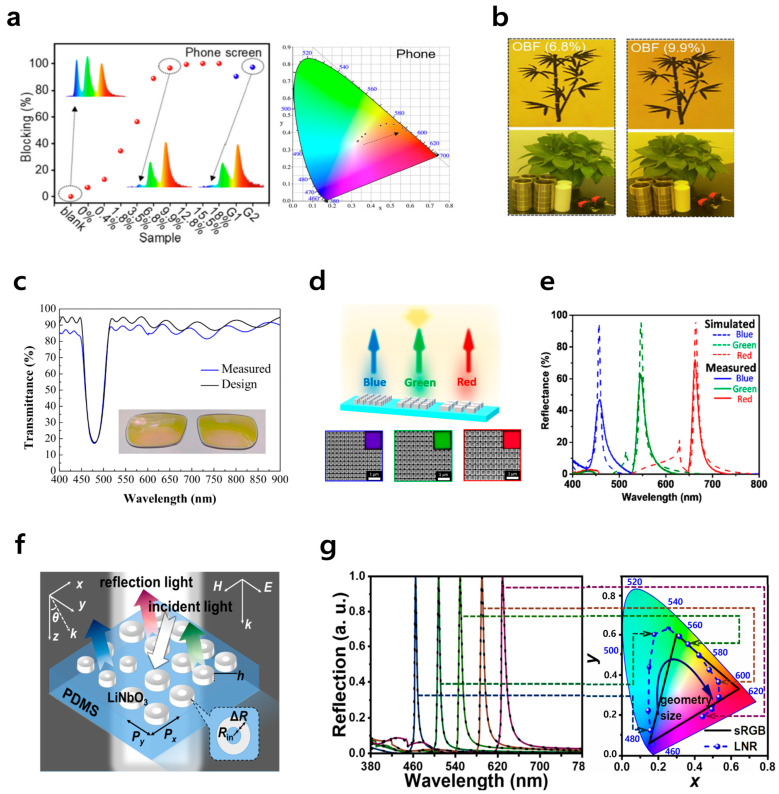
Reduction in blue light using optical filters. (**a**) Dependence of the blue light blocking spectra on Bio-CD concentration (left) and the corresponding shift in CIE chromaticity coordinates from the white towards the yellow/orange region (right). In the spectra, color indicates wavelength across the visible range. Reprinted from Ref. [125] with permission from Elsevier (Academic Press imprint). (**b**) Images of bamboo viewed through OBFs (6.8% and 9.9% Bio-CDs). Reprinted from Ref. [125] with permission from Elsevier (Academic Press imprint). (**c**) Blue light reduction through a notch filter. Reprinted from Ref. [126] with permission from MDPI. (**d**) Pixel design controls the reflected color gamut using the lattice resonance of a silicon nitride metasurface. Reprinted from Ref. [132] with permission from American Chemical Society. (**e**) Reflectance spectra from R, G, and B pixels presented in panel (**d**). Reprinted from Ref. [132] with permission from American Chemical Society. (**f**) Visible metamaterial using lithium niobate nanoring for stretchable color sensing. Reprinted from Ref. [133] with permission from American Chemical Society. (**g**) Reflection spectra and CIE 1931 color coordinates of LNR metamaterial using lithium niobate (LiNbO_3_) nanoring (LNR) structure. In the CIE diagram, the colored background shows the visible gamut. Reprinted from Ref. [133] with permission from American Chemical Society.

Additional approaches exist to passively attenuate harmful blue light externally through the use of optical filters. For example, a technology has been commercialized in which dyes or pigments are coated on display panel surfaces to selectively absorb specific short-wavelength light and convert it into heat for dissipation [122,123,124]. Alternatively, biomass-derived carbon dots (Bio-CDs) enable the creation of optical blocking films in which the degree of blue light filtering can be tuned by controlling their concentration, thereby effectively protecting eyes from harmful blue light, as presented in Figure 6a,b [125]. Another technology involves re-emitting longer-wavelength light through photoluminescence via fluorescent or quantum dot (QD) conversion sheets, thereby achieving high optical efficiency inside the panel. This method can alleviate the blue light intensity without significantly altering the existing devices, making it a commonly used solution. Furthermore, by employing technologies such as dielectric multilayer filters or metasurfaces utilizing optical interference, precise narrowband spectrum control that filters specific light ranges can be achieved. Interference films such as dichroic filters, notch filters, and Bragg reflectors can be used to selectively eliminate the harmful blue light spectrum [126,127,128,129]. Figure 6c shows that the 9-layer optical notch filter composed of SiO_2_ and Nb_2_O_5_ suppresses transmission at the center wavelength of 480 nm. Moreover, controlling the magnetic dipole and lattice resonances of nanostructure arrays enables the suppression of undesired modes and the realization of sharp single-color reflection [130,131,132,133]. Figure 6d,e illustrate the silicon nitride metasurface structure, demonstrating how reflectance wavelength regions, such as blue light, can be precisely controlled by leveraging the lattice resonance phenomenon inherent in this design. Figure 6f,g illustrate a visible metamaterial implemented using a lithium niobate nanoring structure on a stretchable PDMS substrate, demonstrating precise spectrum control through magnetic dipole and surface lattice resonance. Although metamaterial-based designs have not yet been commercialized, they are theoretically capable of achieving high-precision wavelength blocking and are considered to have potential for future applications in display filters. Table 2 summarizes hardware-based solutions aimed at mitigating harmful blue light.

From a software perspective, methods to reduce harmful blue light wavelengths for human health can be categorized into color temperature adjustments, the use of metamerism, light intensity control, and circadian lighting.

**Figure 7:**
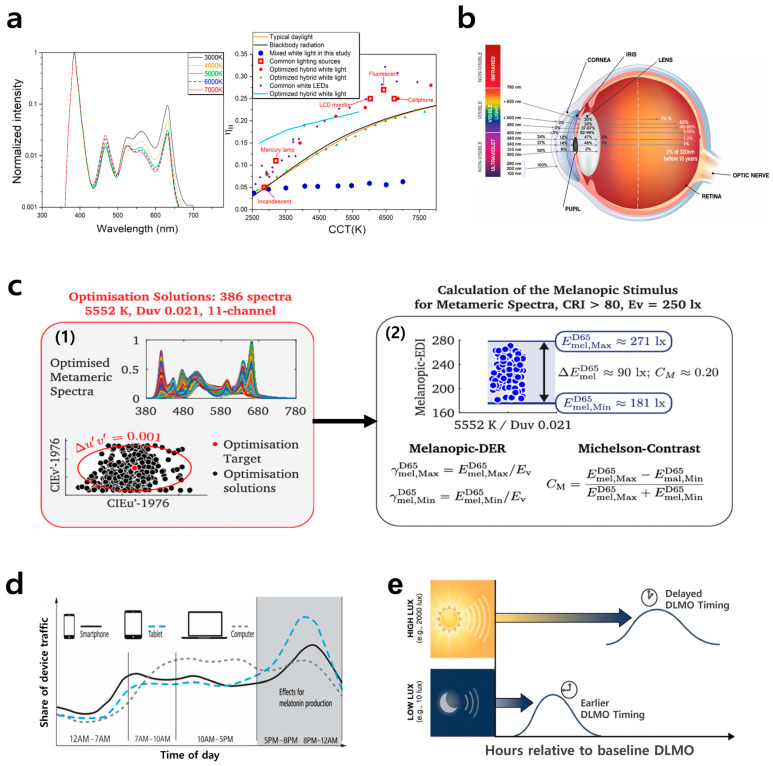
Software-based approaches to diminish harmful blue light, including parameter tuning and human-centric lighting control. (**a**) Electroluminescence spectra (left) and the resulting reduction (right, indicated by blue dots) in blue light hazard efficiency of radiation (BLHER) achieved by adjusting the color temperature and optimizing the mixing ratio of strategic short-wavelength LEDs. Reprinted from Ref. [135] with permission from Elsevier. (**b**) The quantitative ratio of light within a specific wavelength range reaching the retina after transmission through the cornea and lens. Reprinted from Ref. [136] with permission from Springer Nature. (**c**) Identical visual perception of color and brightness despite differing spectral power distributions, resulting from equal stimulation of cone cells (1). Differing levels of ipRGC stimulation by various spectra, as indicated by melanopic metrics (2). Blue dots denote melanopic stimulus values across metameric spectra at the target chromaticity. Reprinted from Ref. [142] with permission from Nature Portfolio. (**d**) Delayed melatonin secretion and disruption of circadian rhythm regulation mechanisms due to increased use of digital devices during a typical workday. Reprinted from Ref. [146] with permission from Wiley-VCH. (**e**) Evening light intensity-dependent delay of Dim Light Melatonin Onset (DLMO). Higher evening light intensity delays melatonin onset; dim light causes less delay and an earlier onset compared with bright light.

A common way to reduce blue light by adjusting the color temperature is to modify the display’s red–green–blue (RGB) ratio, specifically, by lowering the blue component while raising the red and green components [134,135]. Consequently, the display takes on a warm yellowish hue, which can help alleviate eye strain and reduce the suppression of melatonin secretion at night, as shown in Figure 7a. A method for reducing the absolute intensity of harmful light through duty cycle control has also been proposed [136,137,138]. Figure 7b, which quantitatively illustrates the wavelength-dependent light transmission to the retina, suggests that reducing the intensity of specific wavelengths is an effective approach to limiting the amount of blue light that reaches the retina. In LCDs or micro-LED displays that use fast-response LED backlighting, total exposure to short-wavelength blue light can be reduced by lowering screen brightness through PWM-based (pulse width modulation) driving. However, flicker issues may arise in this case, so it is necessary to consider increasing the switching frequency or fine-tuning the current, in combination with hybrid dimming techniques, to enhance ergonomic safety.

In addition to these parameter-tuning methods for mitigating harmful blue light, there are also human perception and physiology-based methods. A proactive approach entails reducing harmful blue light through the application of a metamerism algorithm [139,140,141,142,143,144,145]. For example, if a small amount of red and green light is mixed with a strong peak in the blue region around 460 nm to match the tristimulus values, the human visual system will ultimately perceive a color similar to deep blue, around 420 nm. In this process, the actual amount of short-wavelength light is reduced, yet the visual color change remains minimal, allowing for both blue light reduction and the simultaneous maintenance of image quality. Figure 7c illustrates that even when different spectra appear identical in color and brightness, they can still produce significantly different levels of non-visual melanopsin stimulation. Finally, Figure 7d,e indicate that a user-customized circadian lighting approach, which dynamically adjusts the display’s brightness, color temperature, and spectrum according to different times of day and activity contexts, can minimize the harmful effects of blue light [146,147,148,149,150,151,152]. This strategy integrates biological signals, such as sleep patterns, heart rate, and oxygen saturation, to promote melatonin secretion at night and maintain alertness during the day. As a result, this method improves sleep quality, enhances daytime focus and productivity, and provides preventive and supportive benefits for mood disorders such as seasonal affective disorder. Advanced data collection, modeling based on an understanding of circadian rhythms, personalized intervention algorithms, and engaging UI/UX design are essential for effective digital therapeutic devices. The software-based approaches for mitigating harmful blue light, explained above, are summarized in Table 3.

## 5. Advances and Outlook for Display-Enabled DTx to Support Clinical Efficacy

In the display industry, spectrum design technologies are being developed to minimize the impact of wavelengths between 415 nm and 450 nm on retinal tissue, reducing the risks of ocular diseases such as age-related macular degeneration (AMD) and photochemical damage [153]. These technologies include the methods described earlier, such as modifications to light-emitting materials and structures, optical cut filters, and adjustments to the spectral characteristics of LED chips. However, the goal of short-wavelength light blocking should not be limited to preventing physical damage; it must also consider its impact on the overall biological rhythm [154]. The 450–480 nm spectral band has historically been overlooked, largely due to the perception that it poses relatively minimal direct harm to biological tissues; however, wavelengths in this range can suppress melatonin secretion and disrupt the biological rhythm. Melatonin is not just a hormone responsible for inducing sleep; it plays a crucial role in maintaining the balance of the biological clock, endocrine system, and nervous system, making it an essential hormone. Therefore, technological strategies to prevent the cascade of physiological consequences caused by melatonin suppression—such as poor sleep quality, impaired emotional and cognitive function, weakened immunity, metabolic disorders, and increased risk of cardiovascular diseases—are essential. As digital therapeutics become more widespread, the overall impact of blue wavelengths on the disruption of the biological clock must be reconsidered. The goal of blue light filtering technology is to integrate the consideration of hormone regulation and neural stability, requiring solutions that reduce exposure to wavelengths between 450 and 480 nm to minimize disruption of melatonin secretion. When developing digital therapeutic applications, it is essential to analyze the impact of blue light across different wavelength ranges based on medical objectives. This should involve dynamic adjustment of color temperature according to the usage environment and time of day, color reconstruction algorithms utilizing metamerism, and conditional variable filter technologies, all of which must be applied synergistically and organically. In addition to the application of high-clarity and high-brightness displays, the development of digital therapeutics utilizing extended reality (XR) technologies such as VR and AR may significantly increase blue wavelength exposure as the distance between the display and the eyes decreases [155]. Therefore, considering the development trends in digital therapeutics from the perspective of display technology, the significance of blue light reduction technologies will continue to increase.

Since digital therapeutics are inherently software prescriptions, they offer scalability; however, there are inherent limitations in managing or mitigating unintended side effects when they arise. These characteristics increase the likelihood of unintended risks spreading widely due to the use of DTx, which can lead to serious issues related to patient safety. Therefore, to prevent potential harm caused by blue light exposure in the 450–480 nm wavelength range from displays, clear and specific guidelines must be established and strictly followed throughout the prescription and use of digital therapeutics. Furthermore, when developing displays for digital therapeutics, international standards, regulations, and certification approaches should be advanced to bound blue-light-related exposure under clinically deployed use and to encourage consistent implementation across both hardware and software design. The content discussed in this review provides a structured framework that links wavelength-dependent risk domains to actionable mitigation options, helping inform display design considerations in DTx and highlighting priorities for future validation.

To place these considerations in context, prior blue-light literature has mainly emphasized retinal photochemical hazard and general lighting or consumer-device exposure, while the therapeutic context of display-enabled DTx has been less explicitly addressed. The added value of this review is that it integrates wavelength-dependent risk domains with the DTx clinical setting and links them to actionable mitigation at device and software levels. Limitations include heterogeneous evidence across indications and insufficiently standardized reporting of spectral and temporal parameters, which constrains cross-study comparability. Key gaps remain in DTx-specific real-world quantification of melanopic exposure, long-term outcomes under repeated near-eye XR exposure, and consensus on clinically grounded display specifications. Future work should prioritize harmonized measurement/reporting, exposure–response characterization relevant to therapeutic dosing, and guidance aligning display output with therapeutic intent while preserving visual performance and usability.

## 6. Conclusions

Digital therapeutics, which are new software-based medical technologies with clinical efficacy proven through scientific evidence, are being gradually prescribed in healthcare institutions worldwide. This review examined the harmful effects of blue light from displays, which could interfere with digital therapeutic goals and undermine their clinical effectiveness, and explored potential technological directions to mitigate these effects. Digital therapeutics is a medical intervention that integrates digital devices, such as displays, with psychology, medicine, behavioral science, and communication technologies to deliver therapeutic effects. Therefore, fundamental technologies for modulating or filtering harmful light wavelengths in alignment with digital therapeutic goals are essential, and prescription guidelines incorporating optimized display specifications must be established. In particular, advancements in display technology aimed at minimizing blue light in the 450–480 nm wavelength range, known to inhibit melatonin secretion, can mitigate adverse effects on sleep, cognition, mood, and metabolic health, thereby enhancing the efficacy of digital therapeutics.

## Figures and Tables

**Table 1:** Summary of DTx clinical benefits and potential blue-light-related adverse effects.

Category	Effectiveness of DTx	Ref.	Potential Adverse Effects Associated with DTx Use	Refs.
Sleep	Insomnia Sleep apnea Nightmare	[23,24,25] [26] [27]	Sleep deprivation Prolonged sleep latency Delayed sleep phase syndrome	[65,66,67] [68,69] [70,71]
Mental disorders	Depression Generalized anxiety disorder Social anxiety disorder	[28,29,30] [31,32] [33,34]	Stress Depression Bipolar disorder	[72] [73,74] [75,76]
Cognition and learning	Neurodegenerative diseases Nerve-damage-induced neurological disorders ADHD	[35,36,37] [38,39] [40,41]	Impairments in spatial learning Memory decline Neurodegenerative disease	[77] [78,79] [80,81]
Cardiovascular	Blood pressure regulation Cardiorespiratory function Acute coronary syndrome	[42,43,44] [45,46,47] [48]	Blood pressure changes Cardiovascular dysfunction Heart rate variability	[82] [83,84,85] [86,87]
Metabolism	Diabetes Obesity Hepatic disease	[49,50] [51,52] [53,54]	Gut dysbiosis Hyperglycemia Obesity	[88,89] [90,91] [92,93]
Cancer	Prevention Advanced cancer Post-treatment management	[55] [56] [57,58,59]	Liver cancer Thyroid cancer Skin tumor Prostate cancer Breast cancer	[95] [96] [97] [98,99] [100,101,102]
Skin	Atopic dermatitis Psoriasis Skin rash	[60,61,62] [63] [64]	Pigmentation Skin cell apoptosis Melanoma	[103,104,105] [106] [107]

**Table 2:** Summary of hardware-level strategies to mitigate blue-light exposure during display-based DTx delivery.

Category	Technology	Principle	Refs.
Display light source	OLED	Elemental substitution in organic emitters and optimizing doping, structure, and arrangement	[108,109,110,111,112,113]
	Micro-LED	Epitaxial control of semiconductor bandgap and active layer nanostructure	[114,115,116,117]
	QLED	Tuning quantum dot size, composition, and structure via quantum confinement	[118,119,120,121]
Optical filter	Light absorption and conversion	Light absorption, tunable filtering, and PL conversion using functional materials	[122,123,124,125]
	Dielectric multilayer films	Dichroic, notch, and bragg filters for precise spectral manipulation	[126,127,128,129]
	Metasurfaces/Metamaterials	Resonance engineering for narrowband wavelength-selective filtering or reflection	[130,131,132,133]

**Table 3:** Summary of software-level mitigation strategies for blue-light-related physiological risk in DTx use.

Category	Technology	Principle	Refs.
Parameter adjustment	Color temperature control	Modification of display color balance via RGB ratio tuning	[134,135]
	PWM dimming	Modulating brightness via duty cycle control	[136,137,138]
Human-centric lighting control	Metamerism	Algorithmic generating metamers for perceptual color matching	[139,140,141,142,143,144,145]
	User-customized circadian lighting	Biologically informed dynamic adjustment of display output for circadian alignment	[146,147,148,149,150,151,152]

## Data Availability

No new data were created or analyzed in this study. Data sharing is not applicable to this article.
